# Association between arteriovenous access flow and ventricular function: A cross-sectional study

**DOI:** 10.1016/j.amsu.2022.103649

**Published:** 2022-04-19

**Authors:** Rachmat Ageng Prastowo, Johanes Nugroho Eko Putranto, Iswanto Pratanu, Ryan Enast Intan, Firas Farisi Alkaff

**Affiliations:** a Department of Cardiology and Vascular Medicine, Faculty of Medicine Universitas Airlangga – Dr. Soetomo General Academic Hospital, Jl. Mayjend Prof. Dr. Moestopo No 4-6, Surabaya, East Java, 60286, Indonesia; b Division of Pharmacology and Therapy, Department of Anatomy, Histology, And Pharmacology, Faculty of Medicine Universitas Airlangga, Jl. Mayjend Prof. Dr. Moestopo No 47, Surabaya, East Java, 60132, Indonesia; c Division of Nephrology, Department of Internal Medicine, University Medical Center Groningen, Hanzeplein 1, 9713, GZ, Groningen, the Netherlands

**Keywords:** Arteriovenous anastomosis, Hemodialysis, Left ventricular function, Right ventricular function

## Abstract

**Background:**

Permanent hemodialysis access comes with a myriad of problems on top of the well-known benefits; flow disturbances, risk of infection and revision being among them. All of these could eventually lead to impaired cardiac function. Even so, the relationship between impaired cardiac function due to arteriovenous access in patients undergoing hemodialysis has not been clearly described. This study aimed to analyze the relationship of flow in an artificial arteriovenous access with left and right ventricular function in patients with chronic kidney disease (CKD) undergoing hemodialysis at a referral hospital in Indonesia.

**Material and methods:**

This was a cross sectional study with consecutive sampling technique. Samples were patients with CKD undergoing hemodialysis at Dr. Soetomo General Hospital from December 2021to January 2022. A total of 47 patients who met the inclusion criteria underwent Doppler ultrasound to assess arteriovenous access flow and transthoracic echocardiography to assess left and right ventricle function.

**Results:**

From 47 patients, 26 (55.3%) had high arteriovenous access flow. The clinical characteristics of the patients between the high and low arteriovenous access flow groups were not significantly different. We found that the value of left ventricular ejection fraction in the non-high-flow access group was significantly higher than the high-flow access group (p < 0.05). Other than that, the median right ventricle fractional area changes in the non-high-flow access group was also higher than the high-flow access group (p < 0.05).

**Conclusion:**

Arteriovenous access flow as measured by Doppler ultrasonography has a significant relationship with impaired left and right ventricular functions based on systolic function parameters from echocardiography.

## Highlights

• There is an association between arteriovenous access flow and ventricular function.
• The higher the access flow, the lower the left ventricular systolic function.
• The higher the access flow, the lower the right ventricular systolic function.
• It is important to periodically evaluate the access flow of hemodialysis patients.

## 1 Introduction

Chronic kidney disease (CKD) and cardiovascular disease (CVD) has been recognized as a leading public health problem worldwide. The global estimated prevalence of CKD is 13.4% (11.7–15.1%), and patients with end-stage kidney disease (ESKD) needing renal replacement therapy is estimated between 4.902 and 7.083 million [1]. Meanwhile, an estimated 18.6 million people died from CVDs in 2019, representing the leading cause of all global deaths world-wide [2]. There are known strong correlation between CKD and cardiovascular disease [3]. Creating optimal care for CKD patients requiring hemodialysis continues to be a challenge for nephrologists, cardiologists, and vascular surgeons. It is known that temporary dialysis catheters used in majority of patients initiating dialysis can be a source of intravascular infection and are associated with premature death in CKD patients [4]. Therefore, there is an emphasis on the urgency of establishing access for permanent hemodialysis through surgery, namely arteriovenous fistula (AVF) or arteriovenous graft (AVG) to reduce the patient's exposure and duration of using a dialysis catheter [5].

Permanent hemodialysis access, in addition to providing benefits, also brings a series of problems, such as flow disturbances, risk of infection, and the need for revision [6]. It will also affect the hemodynamic system. AVF or AVG will expose a low pressure and high capacitance venous system to the high pressure and low capacitance arterial system. This will decrease total systemic vascular resistance while increasing venous return to the heart. The counterregulatory response then proceeds with an increase in cardiac output mediated by the sympathetic nervous system and circulating catecholamines. In the initial phase, the heart increases cardiac output by increasing heart rate and stroke volume. Over time, the overstimulation will lead to left ventricular hypertrophy, decreased left ventricular ejection fraction (LVEF), and ultimately heart failure [7].

The relationship between impaired cardiac function due to arteriovenous access in patients undergoing hemodialysis has not been clearly described. Heart failure itself is a comorbidity in hemodialysis patients, and up to half of all deaths in the hemodialysis population are due to CVD [8]. Despite the physiological differences, it is difficult to recognize and diagnose a cardiac dysfunction secondary to AVF, because the signs and symptoms are generally very similar.

Impaired cardiac function can present a difficult treatment dilemma. Ideally, clinicians should treat symptoms and prevent the development of heart failure while simultaneously maintaining adequate vascular access for hemodialysis. Previous studies have showed that there were significant correlation between artificial hemodialysis access flow with cardiac function in CKD patients undergoing hemodialysis. However, the studies are sparse and never been done in Indonesia before [9,10]. Elucidating the relation between the two factors will help provide guidance in determining further therapy and management of CKD patients undergoing hemodialysis using artificial arteriovenous access in compatible population. Therefore, this study aimed to analyze the relationship of flow (Qa) on artificial arteriovenous access (AVF and AVG) with left and right ventricular function in CKD patients undergoing hemodialysis at a referral hospital in Indonesia.

### 1.1 Methods

This was a cross sectional study with consecutive sampling technique. Sampling was carried out on CKD patients undergoing hemodialysis who met the inclusion criteria at Dr. Soetomo General Hospital from December 2021 to January 2022. The inclusion criteria in this study were patients diagnosed with CKD and undergoing hemodialysis with AVF and AVG who were >18 years old and were willing to follow the study procedure by signing an informed consent. The exclusion criteria were CKD patients who had comorbid conditions, such as structural heart disease, hyperthyroidism, or pregnancy.

The independent variable in this study was Qa from AVF and AVG measured using color Doppler ultrasound examination, categorized into two group: high flow group (Qa > 2000 ml/min) and non-high flow (Qa < 2000 ml/min). The outcome of this study was echocardiographic parameters of left and right ventricular function which include: (1) left ventricular systolic function using the teich and biplane method, (2) left ventricular diastolic function, (3) tricuspid annular plane systolic excursion (TAPSE), and (4) right ventricular fractional area change (FAC). For secondary analysis, we analyzed the effect of the Qa/cardiac output (CO), maximal tricuspid regurgitation velocity (TRVmax)/CO, and maximal tricuspid regurgitant pressure gradient (TRmaxPG)/CO ratio index against hyperdynamic condition using cardiac index (CI) parameters of more than 3.9/min/m2 [11].

### 1.2 Ethical statement

This study was conducted in accordance with the Declaration of Helsinki and reported in line with the STROCSS 2021 criteria [12]. Prior to study initiation, approval by the Institutional Ethics Committee of Dr. Soetomo General Hospital has been received (0326//KEPK/XII/2021). This study has also been registered at the Research Registry (www.researchregistry.com) (Unique Identifying Number: researchregistry7793) [13]. All subjects gave their informed consent prior to their inclusion in the study. All data that could reveal the identity of the subjects have been omitted.

### 1.3 Statistical analysis

Statistical analysis was performed using IBM SPSS Statistics version 16.0 (IBM Corp., Armonk, New York) to determine the relationship between Qa and left and right ventricular function, and CI. Ordinal and nominal data will be displayed with frequency and percentage, while interval or ratio data will be displayed by mean ± standard deviation (SD) for normal distributed data, and median and inter quantile range (IQR) for non-normal distributed data. Independent sample *t*-test or Man-whiney was used for comparison between high flow group and non-high flow group where appropriate according to data distribution. The Chi-Square test or Fisher's Exact test where appropriate, was performed to determine associations. Pearson or spearman test was used to analyze correlation between the variables as needed. Two-tailed P value < 0.05 was considered statistically significant.

### 1.4 Patient and public involvement

Patients and the public were not involved in this study.

## 2 Results

### 2.1 Baseline characteristic

A total of 47 patientsmet the inclusion criteria and underwent Doppler ultrasound examination to assess Qa and transthoracic echocardiography to assess left and right ventricle function. Baseline characteristic of this study includes gender, age, body height, body weight, history of hypertension, history of diabetes mellitus, history of stroke, history of hemodialysis and arteriovenous access location.

There were more female patients included in this study(29 patients, 61.7%), with an average age of 47.4 ± 10.8 years, weight 57.1 ± 11.2 kg, and height 159.2 ± 7.5 cm. The most common comorbidity found was hypertension (87.2%). The average duration of history of hemodialysis was 4.6 ± 3.5 years, with the majority of arteriovenous access located on lower arm (53.2%). It was also found that 26 (55.3%) subjects had high Qa (Table 1).

**Table 1:** Baseline Characteristics of study population.

Variables		N(%) or *Mean* ± SD
Gender	Man	18 (38.3%)
	Woman	29 (61.7%)
Age (year)		47.45 ± 10.804
Body height (Cm)		159.28 ± 7.523
Body weight (Kg)		57.15 ± 11.248
History of hypertension	No	6 (12.8%)
	Yes	41 (87.2%)
History of diabetes mellitus	No	38 (80.9%)
	Yes	9 (19.1%)
History of stroke	No	46 (97.9%)
	Yes	1 (2.1%)
Duration of hemodialysis history (year)		4.6 ± 3.53
Arteriovenous access location	*Lower arm*	25 (53.2%)
	*Upper arm*	22 (46.8%)
Arteriovenous access flow (Qa)	High flow (≥2000 ml/min)	26 (55.3%)
	Non-high flow (<2000 ml/min)	21 (44.7%)

### 2.2 Analysis of relationship between baseline characteristics with Qa

An analysis of the basic characteristic variables was performed on Qa to compared between the high flow and non-high flow (Table 2). The results showed that there was no significant difference on all baseline characteristics of gender, history of hypertension, history of diabetes mellitus, history of stroke, age, and duration of hemodialysis history (All p > 0.05), which mean Qa was not influenced by baseline characteristics differences.

**Table 2:** The comparison between the variables of clinical baseline characteristics based on arteriovenous access flow (Qa) group.

Variables		Qa		Total	*P value*
		High flow (n = 21)	Non-high flow (n = 26)		
Gender	Man	7 (38.9%)	11 (61.1%)	18 (38.3%)	0.743^a^
	Woman	14 (48.3%)	15 (51.7%)	29 (61.7%)	
History of hypertension	No	2 (33.3%)	4 (66.7%)	6 (12.8%)	0.678^a^
	Yes	19 (46.3%)	22 (53.7%)	41 (87.2%)	
History of diabetes mellitus	No	18 (47.4%)	20 (52.6%)	38 (80.9%)	0.711^a^
	Yes	3 (33.3%)	6 (66.7%)	9 (19.1%)	
History of stroke	No	21 (45.7%)	25 (54.3%)	46 (97.9%)	1.000^a^
	Yes	0 (0.0%)	1 (100.0%)	1 (2.1%)	
Age (year, mean ± SD)		50.76 ± 12.012	44.77 ± 9.092		0.058^b^
Duration of hemodialysis history (year median, IQR)		3.00 (1–15)	3.00 (1–11)		0.795^c^

a *chi-square test*.

b independent T-test.

c Mann Whitney test.

### 2.3 Analysis of the relationship of Qa with left ventricular systolic function

**Fig. 1:**
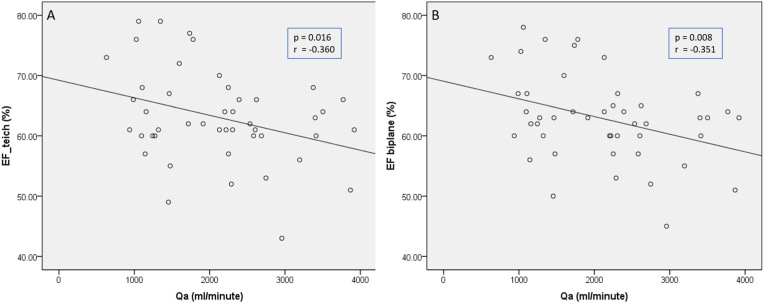
Scattered plot between Qa and EF A) by teich, B) by biplane.

Left ventricular systolic function analysis was performed with the variables EF Teich, EF Biplane, CO, and CI based on the Qa category (Table 3). The results showed that both EF by Teich and EF by biplane in the non-high flow access group was significantly higher than in the high flow access group (p value 0.012 and 0.02 respectively). Meanwhile, there were no significant difference of CO and CI between non-high flow and high flow access group (*p value* 0.296 dan 0.697 respectively). Further analysis with pearson and spearman correlation also confirmed this finding by showing significant negative moderate correlation between Qa and EF either by Teich and by biplane with Qa (p = 0.016, r = −0.360 and p = 0.008, r = −0.351 respectively) (Fig. 1).

**Table 3:** Association between arteriovenous access flow (Qa) and left ventricular functional parameters.

	Qa				P value
	Non-high flow (n = 21)		High flow (n = 26)		
	Range	Mean ± SD/Median (IQR)	Range	Mean ± SD/Median (IQR)	
EF Teich	50.00–78.00	65.90 ± 8.47	36.00–73.00	59.85 ± 7.47	0.012^a^
EF Biplane		64.00 (57.00–71.00)		61.00 (47.00–67.00)	0.020^b^
CO		5.60 ± 1.36		5.98 ± 1.16	0.296^a^
CI		3.67 ± 1.01		3.78 ± 0.83	0.697^a^

CI = cardiac index; CO = cardiac output; EF = ejection fraction.

a Independent T-test.

b Mann Whitney test.

### 2.4 Qa analysis with left ventricular diastolic function

Next, the diastolic function (E/e', E/A ratio, left atrial volume index [LAVI]) test was compared based on the Qa category. The results showed that the E/e' in the non-high flow group was lower than the high flow group, however the difference was not statistically significant (p value = 0.920). Meanwhile, the median results of E/A and LAVI were relatively similar in the low-flow group compared to the high-flow group, which means there was no significant relationship between E/A and LAVI with Qa (p value 0.748 and 0.991 respectively) (Table 4).

**Table 4:** Association between arteriovenous access flow (Qa) and left ventricular diastolic function parameters.

	Qa				P value
	Non-high flow (n = 21)		High flow (n = 26)		
	Range	Mean ± SD/Median	Range	Mean ± SD/Median	
E/e’		12.56 ± 4.47		12.70 ± 4.63	0.920^a^
E/A	0.61–3.32	1.07	0.57–2.74	1.03	0.748^b^
LAVI	10.56–81.91	31.02	13.09–59.20	30.56	0.991^b^

a Independent T-test.

b Mann Whitney test.

The diastolic function was then categorized based on the grades and compared between the two Qa groups. The calculation results showed that grades 1 and 2 diastolic dysfunction were more common in high flow conditions than low flow conditions, however the difference was not statistically significant, so Qa did not determine the degree of left ventricular diastolic dysfunction severity (p value > 0.05) (Table 5).

**Table 5:** Cross tabulation of arteriovenous access flow (Qa) group with the degree of left ventricular diastolic dysfunction.

Left ventricular diastolic function	Qa		Total	Chi-square P value
	Non-high flow (n = 21)	High flow (n = 26)		
Normal	8 (50.0%)	8 (50.0%)	16 (34.0%)	0.617
Grade I	6 (42.9%)	8 (57.1%)	14 (29.8%)	
Grade 2	7 (41.2%)	10 (58.8%)	17 (36.2%)	
Total	21 (44.7%)	26 (55.3%)	47 (100.0%)	

### 2.5 Analysis of Qa relationship with right ventricular function

**Fig. 2:**
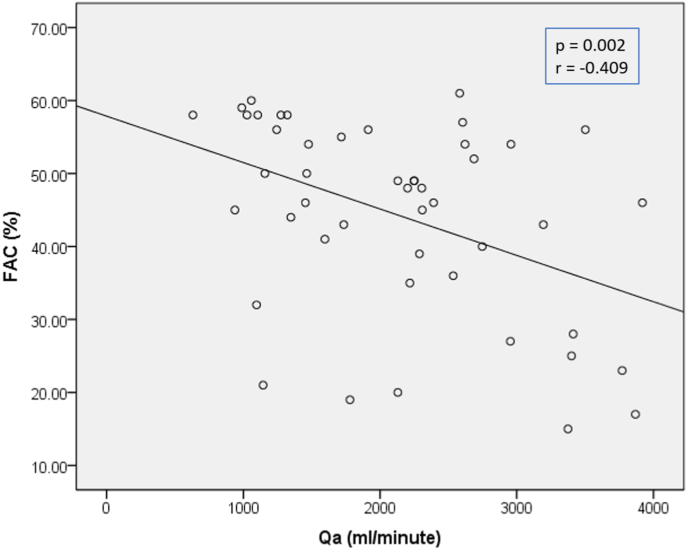
Scattered plot between Qa and FAC.

Next, the TAPSE & FAC analysis test were carried out based on the Qa category. The median results showed that the TAPSE value was relatively the same in the low flow group compared to the high flow group (p value = 0.331). Meanwhile, FAC was influenced by the Qa category, proved by the result of the median FAC value in the non-high flow access group which was significantly higher than the high flow group (p value = 0.022) (Table 6). Further analysis with spearman correlation test also showed significant negative moderate correlation between Qa and FAC (p = 0.002, r = −0.409) (Fig. 2).

**Table 6:** Comparison of right ventricular systolic function based on arteriovenous access flow (Qa) group.

	Qa				
	Non-high flow (n = 21)		High flow (n = 26)		
	Range	Median	Range	Median	Mann Whitney P value
TAPSE	1.30–2.40	2.00	1.40–2.50	1.95	0.311
FAC	19.00–60.00	54.00	15.00–61.00	45.50	0.022

FAC = fractional area change; TAPSE = tricuspid annular plane systolic excursion.

### 2.6 Ratio index analysis on hyperdynamic conditions

A description of several ratio indices, i.e., Qa/CO, TRVmax/CO, and TRmaxPG/CO, were performed based on the category of hyperdynamic conditions using CI parameters of more than 3.9/min/m2 [11]. The results of descriptive calculations showed that the mean values of Qa/CO, TRVmax/CO, and TRmaxPG/CO in the non-hyperdynamic group were higher than the hyperdynamic group although it did not reach the statistical significance (Table 7).

**Table 7:** Descriptive value of Qa/CO, TRVmax/CO & TRmaxPG/CO based on CI.

	CI ≤ 3.9 l/min/m^2^ (N = 28)			CI > 3.9 l/min/m^2^ (N = 19)			P value
	Range	Mean ± SD	Median	Range	Mean ± SD	Median	
Qa/CO	0.13–0.76	0.41 ± 0.16	0.38	0.11–0.56	0.33 ± 0.14	0.33	0.143^a^
TRVmax/CO	0.18–1.23	0.43 ± 0.22	0.36	0.08–0.66	0.37 ± 0.16	0.38	0.615^b^
TRmaxPG/CO	0.64–22.4	4.49 ± 4.56	3.18	0.25–11.1	4.34 ± 3.11	4.25	0.743^b^

CO = cardiac output; Qa = arteriovenous access flow; TRVmax = maximal tricuspid regurgitation velocity; TRmaxPG = maximal tricuspid regurgitant pressure gradient.

a Independent T-test.

b Mann Whitney test.

## 3 Discussion

In this observational analytical study, evaluation of Qa and cardiac function by Doppler ultrasonography and transthoracic echocardiography were performed on a total of 47 CKD patients undergoing hemodialysis. There were 26 patients (55.3%) with a Qa reading of more than 2000 ml/min. These patients were classified as high-flow access and considered to be associated with impaired cardiac function in this study. The basic characteristics of the research subjects which include, age, gender, history of hypertension, history of diabetes mellitus, history of stroke, and duration of hemodialysis history had no significant difference and do not affect the Qa group category from our bivariate analysis.

There was a significant difference, however, in the LVEF. Patients with high flow access had lower mean LVEF than those with low flow access. The correlation test also showed a moderate negative correlation, which means the higher the Qa was, the lower the LVEF. This result is in line with the findings of Saleh et al. (2018), where they found patients in the high-flow access group showed a significantly lower ejection fraction with an average value of 57.32% compared to 62.90% for the low access flow group [9]. Another retrospective cohort study by Reddy et al. observed the results of the echocardiographic parameters of CKD patients who would receive hemodialysis before and after AVF installation at an interval of 4 years. There was a significant decrease in left ventricular ejection fraction after AVF installation compared to before AVF insertion, which may worsen heart failure in CKD patients [10].

Our findings support the data from several studies from the United States and Europe which suggest that the mortality and prevalence of heart failure may be increased in patients with AVF access compared to patients receiving peritoneal dialysis. In AVF, connecting arteries to veins results in wasted blood flow because flow in the fistula does not contribute to tissue perfusion. Cardiac output and ventricular work increase in response to decreased arterial resistance, while venous return to the heart increases substantially, resulting in an increase in volume load. These changes can lead to a high-output heart failure in some patients [10]. For this reason, it can be suggested that the higher the flow in AVF, the higher the chance of reduced LV function. Recent case series reports have also shown that reducing AVF flow in high flow rate setting can improve ventricular remodeling [14].

Unlike the result of the LV systolic function, the results of the statistical analysis in LV diastolic function showed that there was no significant association between Qa and LV diastolic function, either by the parameters of the E/e' ratio, E/A ratio, and LAVI, as well as by using the grading parameters according to the guideline of American Society of Echocardiography (ASE) [15]. For the mean value of the E/e' ratio, E/A ratio, and LAVI in the high-flow and non-high-flow access groups, the results were not much different (p > 0.05). In a total of 47 patients in this study, 14 (29.8%) patients had grade I diastolic dysfunction and 17 (36.2%) patients had grade II. Patients with high flow access dominated the number of patients with diastolic dysfunction with 8 (57.1%) patients with grade I diastolic dysfunction and 10 (58.8%) patients with grade II diastolic dysfunction. This indicated that impaired left ventricular diastolic function with increased left ventricular filling pressures is seen in some, but not all, patients with CKD. Our finding was similar to some previous studies. Panoulas and colleagues found that diastolic dysfunction was more common in CKD stages 3–5 (70%) with grade I diastolic dysfunction (relaxation disorder) suffered by most (40%), followed by grade II (30%) [16]. Another evidence from a prospective observational study of 57 CKD patients undergoing hemodialysis and 96 patients without hemodialysis (mean eGFR 22 0.3 ± 7.4 mL/min/1.73 m2) found diastolic dysfunction in 85% of patients, with 35% exhibiting grade II diastolic dysfunction [17].

In addition to having an impact on the left ventricle, CKD and hemodialysis with arteriovenous access also have an impact on the right ventricle, as the increase in volume load from AVF/AVG flow results in an increase in the right ventricular pressure and volume, which in turn impairs function. Our result showed a significant association between Qa and right ventricular FAC parameters with a moderate negative correlation. There was also a significant difference in the right ventricular FAC value between the Qa group, where the FAC was significantly lower in the high-flow access group compared to the non-high-flow access group. These results are in line with a previous study by Reddy et al. (2017) in which there was a two-to threefold increase in the prevalence of right ventricular dilation and dysfunction after AVF/AVG creation. It may reflect a state of volume overload caused by excessive shunt flow from Arteriovenous access. The previous study demonstrated for the first time that worsening right ventricular dilatation was independently associated with increased mortality and progression of heart failure in patients with CKD undergoing hemodialysis [10].

Basile et al. (2008) found that a limit of Qa > 2000 ml/min and the concept of increased cardio-pulmonary recirculation using a Qa/CO ratio with a limit of >20% could be associated with the risk of high-output heart failure [18]. We performed an analytical sub-study to determine the relationship of the Qa/CO ratio with hyperdynamic conditions described by a CI value of more than 3.9/min/m2, which is one of the parameters of high-output heart failure [19]. In addition, we also looked for the association of a tricuspid regurgitation (TR), which is one of the parameters for assessing volume-load-related pulmonary hypertension with left ventricular CO and CI. TR, which includes functional or mild TR, moderate TR and significant severe TR, is one of the most important functions of the right heart and is very prevalent in various populations The results of statistical tests showed that there was no association between TR Vmax/CO, TR max PG/CO, and Q/CO ratio index with CI in the subjects of this study. This could be due to the insufficient number of subjects to be able to produce a significant relationship.

Since there is a significant relationship between Qa and left and right ventricular functions, we argue that it is then very important to conduct periodic evaluations in CKD patients undergoing hemodialysis. Interventions and medications to reduce ventricular remodeling in the early phase of cardiac dysfunction have the opportunity to prevent more progressive damage due to impaired access and CKD itself. Anti-remodeling drug strategies to surgery to reduce excessive access flow might be an option in the treatment of cardiac dysfunction, and in some studies arteriovenous access modification has a good impact on ventricular remodeling [10].

### 3.1 Limitation

The main limitations of this study are the small number of samples and the cross-sectional study design. Further research with larger study subjects with cohort study design is needed to confirm the findings of this study.

## 4 Conclusion

There is a significant relationship between Qa with left and right ventricular systolic functions using right ventricular FAC parameters in CKD patients undergoing hemodialysis. In contrary, there is no significant relationship between Qa and left and right ventricular systolic functions using the TAPSE parameter in CKD patients undergoing hemodialysis.

## Ethical approval

This study was conducted in accordance with the Declaration of Helsinki and reported in line with the STROCSS 2021 criteria. Prior to study initiation, approval by the Institutional Ethics Committee of Dr. Soetomo General Hospital has been received (0326//KEPK/XII/2021). This study has also been registered at the Research Registry (www.researchregistry.com) (Unique Identifying Number: researchregistry7793). All subjects gave their informed consent prior to their inclusion in the study. All data that could reveal the identity of the subjects have been omitted.

## Sources of funding

This research received no specific grant from any funding agency in the public, commercial, or not-for-profit sectors.

## Author contribution

RAP designed the study, performed data collection, and writing the original draft, JNEP designed the study and writing the original draft, IP designed the study and writing the original draft, REI performed data analysis and revised the draft for important intellectual content, FFA performed data analysis and revised the draft for important intellectual content.

## Registration of research studies

Name of the registry: Research Registry.

Unique Identifying number or registration ID: researchregistry7793.

Hyperlink to your specific registration (must be publicly accessible and will be checked):

https://www.researchregistry.com/browse-the-registry#home/registrationdetails/624e534e3076fe001e912f2d/

## Guarantor

JNEP and IP are the guarantor for this study.

## Consent

All subjects gave their informed consent before their inclusion in the study. All data that could reveal the identity of the subjects have been omitted.

## Provenance and peer review

Not commissioned, externally peer-reviewed.

## Declaration of competing interest

The authors have no conflicts of interest to declare.
